# Cell-in-Cell Structures in the Liver: A Tale of Four E’s

**DOI:** 10.3389/fimmu.2020.00650

**Published:** 2020-05-13

**Authors:** Scott P. Davies, Lauren V. Terry, Alex L. Wilkinson, Zania Stamataki

**Affiliations:** ^1^Centre for Liver and Gastrointestinal Research, Institute of Immunology and Immunotherapy, University of Birmingham, Birmingham, United Kingdom; ^2^NIHR Birmingham Liver Biomedical Research Centre, University Hospitals Birmingham NHS Foundation Trust, Birmingham, United Kingdom

**Keywords:** cell-in-cell, liver, efferocytosis, entosis, emperipolesis, enclysis, cancer, regeneration

## Abstract

The liver is our largest internal organ and it plays major roles in drug detoxification and immunity, where the ingestion of extracellular material through phagocytosis is a critical pathway. Phagocytosis is the deliberate endocytosis of large particles, microbes, dead cells or cell debris and can lead to cell-in-cell structures. Various types of cell endocytosis have been recently described for hepatic epithelia (hepatocytes), which are non-professional phagocytes. Given that up to 80% of the liver comprises hepatocytes, the biological impact of cell-in-cell structures in the liver can have profound effects in liver regeneration, inflammation and cancer. This review brings together the latest reports on four types of endocytosis in the liver -efferocytosis, entosis, emperipolesis and enclysis, with a focus on hepatocyte biology.

## Introduction

*Cell-in-cell* (CIC) structures are formed when a whole cell resides inside the cytoplasm of another, and they have been observed for decades in various contexts. The best characterized CIC mechanism is known as *efferocytosis*, the clearance of dead or dying cells by professional and non-professional phagocytes (1–9). Yet, CIC structures, in which the internalized cell remains viable, have been observed for over a century (10). Recent work has provided evidence for the role of hepatocytes, the principal parenchymal cell within the liver, in several of these processes: *efferocytosis* (1), live cell internalization events including *suicidal emperipolesis* (11), *entosis* (12) and *enclysis* (13) (Table 1). Although the immediate consequences of dead and live cell capture have been investigated, the biological implications and impact on clinical outcomes remain to be elucidated.

**TABLE 1:** The mechanism of cell-in-cell structures.

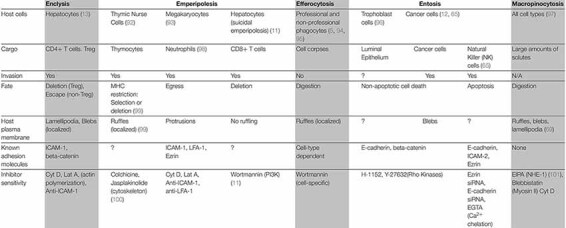

*The best-characterized process is efferocytosis, where multiple receptors have been identified, however, only one receptor is known for hepatocytes (ASGR1). Receptors unique to entosis, emperipolesis or enclysis have not been identified.*

The liver receives 75% of its blood supply from the gastrointestinal tract via the hepatic portal vein (14). As such, it is persistently challenged by toxic substances and microbial- or food-derived antigens. Not only must the liver function to detoxify and neutralize harmful products it is exposed to, it must also maintain an immunotolerising environment so as not to initiate inappropriate immune responses to commensal microbes and food antigens. Nonetheless, the liver must retain the ability to mount a rapid immune response in the case of infection. The role of the liver in immunity is well-established (15), and the cells residing within it are finely tuned to maintain the balance between immunotolerance and immunogenicity. If this balance is perturbed and tolerance is breached, liver disease can develop due to hepatocyte damage during inflammation.

Chronic liver diseases follow a common pathway of progression independently of etiology. Repeated liver injury results in fibrosis, cirrhosis and ultimately, end-stage disease leading to liver failure, the only viable treatment option for these patients is liver transplantation, which is associated with significant pitfalls including organ shortage and graft rejection. Since liver disease continues to increase worldwide (16) there is an unmet clinical need to develop novel therapies that will alleviate chronic inflammation, prevent fibrosis or boost liver immunity in the context of viral infection and primary or metastatic liver cancer. Hepatocytes constitute an attractive target for therapy in these patients, because (i) they are uniquely found in the liver, (ii) they drive regeneration in injury, (iii) they are the focus of infection or malignancy in hepatocellular carcinoma, (iv) they are a natural destination for drug absorption, and (v) unlike targeting immune cells, hepatocyte-directed therapies are unlikely to cause systemic immunosuppression or autoimmunity. We propose that targeting CIC structures has the potential to lead to clinical benefit for patients with liver diseases.

## Efferocytosis

The capture and deletion of dying cells by *efferocytosis* (from *effere*, Latin for “to take to the grave,” “to bury”), a specialized form of phagocytosis, is a crucial process for the liver with important biological impact (1). The liver is inundated with infiltrating immune cells that are destined to die by apoptosis and be digested by liver cells (17). The frequent turnover of hepatocytes, associated with the detoxification of waste products, further contributes to the dead cell burden faced by the liver. Failure to clear these cell corpses can spell disastrous immune consequences, including premature inflammatory responses and an increased risk of autoimmune disease (8).

**FIGURE 1:**
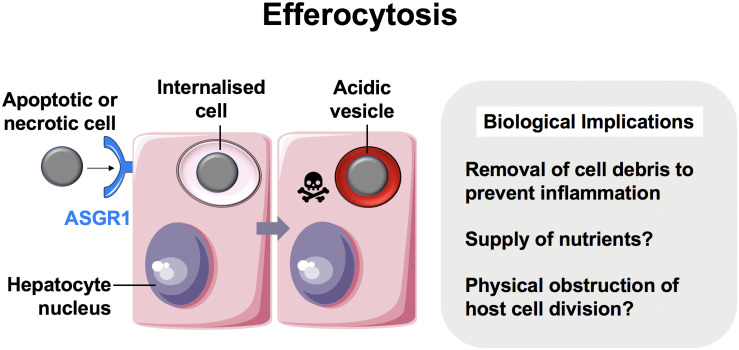
Hepatocytes are important efferocytes. Comprising up to 80% of the liver mass, hepatocytes engulf apoptotic and necrotic cells via the asialoglycoprotein receptor (ASGR1) (3), and rapidly degrade them. This process may supply the liver with nutrients while maintaining homeostasis by eliminating pro-inflammatory cell debris. CIC structures can also lead to failure of cytokinesis (19) and this can impact hepatocyte ploidy.

To prevent the build-up of cellular debris, the cellular composition of the liver is uniquely prepared, it is frequented by monocyte-derived macrophages and possesses a specialized resident macrophage population known as Kupffer cells, which arise following signals from liver-resident cells (9, 18). Aside from these “professional phagocytes,” liver- parenchymal and non-parenchymal cells can also capture and delete dying cells. These “non-professional” populations include hepatic sinusoidal endothelial cells (HSECs), biliary epithelial cells (BECs), stellate cells and hepatocytes (Figure 1) (3, 4, 6, 7). As such, the liver is universally prepared to rapidly clear cell corpses, thus maintaining its immune tolerance.

An astute adaptation of the liver to manage the persisting need to clear dying cells is for its principal cell type, the hepatocyte, to be adept at efferocytosis (1). Hepatocytes are epithelia tasked with drug detoxification and can undergo necrotic cell death in the process, thus neighboring hepatocytes are most likely to make first contact with a dying cell. Hepatocyte efferocytosis was first described in 1952, when Rosin and colleagues observed the presence of erythrocytes within the cytoplasm of hepatocytes (2). This was later ratified by Dini et al., who showed that hepatocytes could also engulf apoptotic cells (3). The same investigation suggested a role for asialoglycoprotein receptor 1 (ASGR1) in the recognition of these cells. Experiments conducted in our group has further confirmed hepatocyte ability to engulf necrotic cells in health and in cancer (1, 13). Other cells which have this capability generally require alternative, more improvisational molecular mechanisms to capture necrotic cells, compared to those known for apoptotic cell capture (5, 20). Hepatocytes can both remove and replenish areas of necrotic sheets associated with disease-related hepatotoxicity, and drive regeneration during injury (21–23).

In contrast to the liver’s professional phagocyte populations, the mechanisms by which hepatocytes clear dead cells are poorly understood. There are few candidate receptors, in addition to ASGR1, by which hepatocytes may recognize and capture dying cells. The consequences of efferocytosis for the hepatocyte have also not been widely explored. The hepatocyte would be granted nutrients from the lysosomal digestion of captured cells. Hepatocytes may also acquire increased genetic diversity at the cellular level through efferocytosis. Efferosomes may physically impede cytokinesis, causing the engulfing hepatocyte to become multinucleate, as seen in breast cancer cells with CIC structures (12). Increased genetic diversity amongst hepatocytes has been shown to increase the ability of the liver to adapt and regenerate in response to a wider variety of insults (24, 25). Efferocytosis may be a mechanism which accelerates this phenomenon, although this may also increase the risk of contracting mutations associated with the onset of hepatocellular carcinoma (HCC). Although hepatocyte multinucleation is both frequent and tolerated in the liver, particularly in older individuals (26, 27), chronic efferocytosis resulting from disease-associated necrosis may promote the acquisition of oncogenic mutations. As the onset of HCC is rarely spontaneous and frequently associated with chronic liver disease, increased hepatocyte efferocytosis may represent a risk factor for its onset.

Dysregulation of efferocytosis in the liver can lead to disease development. This has been exemplified in the case of macrophage clearance, knockout mice lacking hepatic macrophages that express the dead cell scavenger receptor, MerTK, showed exasperated damage when treated with acetaminophen (APAP) (28). More recently it was demonstrated that carbon tetrachloride-treated glycoprotein NMB (gpnmb) KO mice, whose macrophages lack the ability to process internalized cells, showed greater activation of pro-fibrotic myofibroblasts (29). It is also likely that the dysregulation of hepatocyte efferocytosis may contribute to the pathogenesis of other chronic liver diseases. Autoantibodies against ASGR1 have been detected in patients with autoimmune hepatitis (30, 31). Additionally, ethanol-treated rat hepatocytes were shown to be defective in ASGR1-mediated efferocytosis (32).

The effects of aging and the accompanying immune paresis must be considered in liver homeostasis, specifically regarding the clearance of apoptotic and necrotic cells. In both aging and chronic liver disease, there is an accumulation of senescent cells, which produce senescence-associated secretory phenotype (SASP) factors. SASP factors include pro-inflammatory cytokines and growth factors, that have been noted to alter the local microenvironment and induce paracrine senescence and in turn, immuno senescence (33–35). One characteristic of immune senescence is the reduced capacity of a cell to phagocytose, which may contribute to persistent inflammation in older individuals, termed “inflammageing” and lead to defective clearance and resolution of inflammation (36, 37).

Whilst little is understood about hepatocyte efferocytosis in terms of aging, several *in vivo* studies have shown an age-associated decline in macrophage efferocytosis in other tissue types. For example, one study observed that peritoneal macrophages from aged (24-month old) mice had an impaired ability to efferocytose apoptotic Jurkat cells, compared to 2-month old, young mice (38). This result was similarly observed by Linehan et al., whom proceeded to transplant young (8 to 12-week-old mice) peritoneal macrophages into aged (15 to 20-month-old mice) peritoneal space (39). The transplanted, young macrophages in fact exhibited a diminished ability to efferocytose post-transplantation, suggesting that the microenvironment lead to alterations in the efferocytic ability. Additionally, there was a decline in the ability of alveolar macrophages to efferocytose neutrophils in aged mice, which may contribute to lung damage (40). Based on links drawn between diminished efferocytic capacity and old age, it is logical to infer that hepatocytes could be subjected to similar pressures from aging and this warrants further investigation.

Further understanding into the mechanisms of hepatocyte efferocytosis will likely provide opportunities for promoting dead cell clearance and thus preventing immature inflammatory responses in the liver.

## Entosis

**FIGURE 2:**
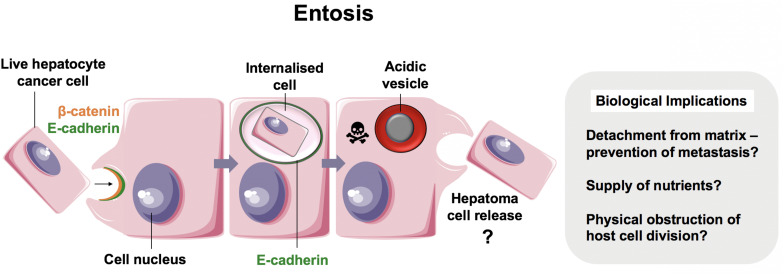
Entosis in neoplastic hepatocytes. We recently showed that hepatocellular carcinoma cells were able to engulf their live neighbors by entosis (13). Entosis is an important disease pathway in cancer epithelia involving E-cadherin and β-catenin (12). Tumor cells that detach from matrix are prone to entosis, and further research is necessary to measure its implications in patients with hepatocellular carcinoma.

For over a century, CIC structures in which viable cells are internalized into other cells have been reported (10, 41, 42). Live cells have been shown to invade or be engulfed by host cells of non-phagocytic origin. Unlike with efferocytosis, which consistently targets cell corpses for lysosomal degradation, these cells can remain viable within vacuole-like structures for long periods and succumb to variable outcomes depending on the context. Although the molecular mechanisms for most examples of live CIC formation generally remain poorly understood, several processes are well-described in the literature. One of these is known as *entosis* (εντóς, inside, into, within) (Figure 2) (41, 43).

In 2007, Overholtzer and colleagues reported that extracellular matrix detachment of cancer cells could promote CIC formation via contractile forces associated with adherens junction formation. This process involved junctional proteins, E-cadherin and β-catenin, and was dependent on actomyosin contractility mediated by Rho-associated coiled-coil-containing protein kinase (ROCK) activity in the target cell specifically (12). This finding, coupled with time-lapse microscopy of CIC formation, was strongly suggestive of target cell invasion as opposed to engulfment and has since been confirmed in several studies (44, 45).

The plasma membrane is the primary site for initiating CIC formation. Plasma membrane blebbing and polarized actin dynamics have been suggested as drivers of entotic invasion (45), with a recent study demonstrating the requirement for the myocardin-related transcription factor-serum response factor (MRTF-SRF) pathway and subsequent sustained ezrin-dependent plasma membrane blebbing (44). Furthermore, in addition to the requirement for adherens junctions (12, 46, 47), studies have shown that the composition of the plasma membrane play a role in entosis. Both liposomes and cholesterol were shown to inhibit CIC formation, presumably by hindering myosin light chain phosphorylation and thus actomyosin contractility (48).

The fate of the internalized cell is variable, most succumb to non-apoptotic cell death and lysosomal degradation, although some target cells occasionally undergo division or release (12, 43, 49–51). Thus, the biological consequences of entosis and the impact on tumor biology remain controversial (52). Since degradation of target cells by neighboring cancer cells has the potential to limit tumor growth, then perhaps entosis represents an intrinsic tumor suppressor mechanism, by which metastatic cancer cells that become detached from matrix are eliminated. Yet, adherent epithelial cells can also undergo entosis, a process driven by mitosis and negatively regulated by cell cycle protein Cdc42 (46). Furthermore, tumor cell cannibalism could promote host cell survival by providing nutrients to those which lack vascular access (53). In support of this, Overholtzer’s group later demonstrated that entosis is induced in adherent cells by glucose starvation, in a manner requiring activity of target cell AMP-activated protein kinase (AMPK) (54). The ability of cancer cells to adapt to starvation by performing entosis and enabling nutrient recovery would confer metabolic advantage of malignant cells, thereby promoting progression of more aggressive tumors. Indeed, it has been proposed that there is direct competition between cancer cells, dictated by mechanical deformability and subsequent entosis, thus ensuring the survival of the most adapted tumor cells (55). These findings highlight the importance of the tumor microenvironment in regulating intracellular signaling pathways that mediate entosis and tumor survival.

The clinical impact of entosis in hepatocellular carcinoma has not been investigated. Similarly to observations made in other epithelial cells, we reported recently that hepatomas cultured in 2D were also capable of engulfing their neighbors (13). The vesicle that housed the internalized cell was enriched in E-cadherin, suggesting that this was another example of entosis (Figure 2). It is not yet clear if non-neoplastic hepatocytes perform entosis. Regardless, liver cancers may benefit from entosis as a source of adaptation and nutrition. Given that there is no effective therapy and the incidence of hepatocellular carcinoma is increasing in the West (56), targeting entosis may prove to be of clinical value.

## Emperipolesis

**FIGURE 3:**
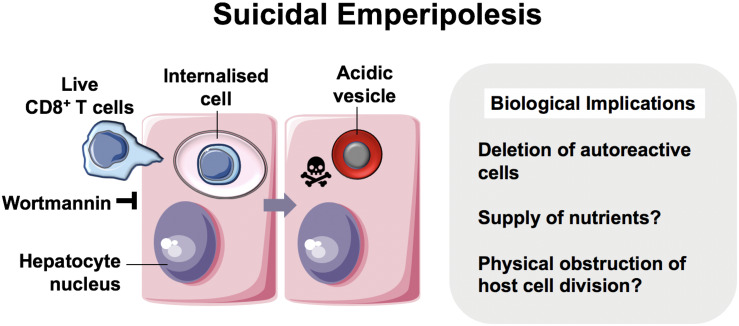
Suicidal Emperipolesis. The seminal work by Benseler et al. provided the first evidence for a biological role of the cell-in-cell structures described as emperipolesis, where immune cells were engulfed alive by hepatocytes (11). In this study, autoreactive CD8^+^ T cells were deleted by suicidal emperipolesis in the liver. The mechanism of capture is not understood, however, perturbation of this process led to breach of liver tolerance in mice.

*Emperipolesis* is a term coined by Humble et al. (57) and used to describe the movement of live cells following internalization (“inside-round-about wandering”) (Figure 3) (57). It has been proposed that whilst CIC and emperipolesis should be used generically to describe the process of cell movement associated with CIC structures, cannibalism and entosis should be used to refer to mechanisms of CIC formation specifically (10, 41). Cell-in-cell structures, or emperipolesis, have long been observed by histopathologists in several types of chronic liver disease. Emperipolesis is increased in autoimmune hepatitis (58, 59) and chronic viral infection (60, 61), suggesting a potential role in liver injury or T cell clearance (62, 63). The precise physiological and pathophysiological role of emperipolesis, however, remains elusive.

The first demonstration of a physiological role for emperipolesis in the liver was reported by Bertolino and colleagues in 2011. They defined a mechanistically distinct type of emperipolesis known as *suicidal emperipolesis*, in which autoreactive CD8^+^ T lymphocytes actively invade hepatocytes and undergo lysosomal degradation (11, 64). Inhibition of this process by wortmannin led to intrahepatic accumulation of autoreactive cells and a breach of tolerance. Wortmannin-treated mice developed immune-mediated hepatitis 3 days post-infusion with autoreactive CD8^+^ T cells, as determined by raised alanine aminotransferase levels and histological liver damage. The authors therefore proposed this as a mechanism of extrathymic regulation for maintaining immune tolerance within the liver.

There is also evidence for a pathophysiological role of CIC structures. *Emperitosis*, another form of emperipolesis, was the name initially given to natural killer (NK) cell invasion of tumor cells and subsequent programmed cell death. Like entosis, emperitosis also requires cadherins, Rho/ROCK proteins and ezrin (65, 66). In contrast to entosis, NK cells succumb to caspase-3-mediated apoptosis, which was attributed to granzyme B accumulation within the vacuole (65). This process has also been extended to human cytotoxic regulatory T cell line, HOZOT, which actively penetrate cancer cell lines but not cells of non-neoplastic origin (67). It is therefore conceivable that emperitosis of cytotoxic immune cells serves as one of the many mechanisms employed by cancer cells to evade immune surveillance. Furthermore, a recent study showing that internalization of anti-fibrotic NK cells in HBV cirrhotic patients is transforming growth factor-β-dependent and may represent a novel mechanism of fibrogenesis (68). Further work is required to fully elucidate the molecular mechanisms of suicidal emperipolesis, which may allow therapeutic targeting in the context of liver transplantation, autoimmune disease and viral hepatitis. Nevertheless, the evidence that this process is distinct from other CIC mechanisms is compelling, and is one example of the complex pathways which can underlie CIC formation.

## Enclysis

**FIGURE 4:**
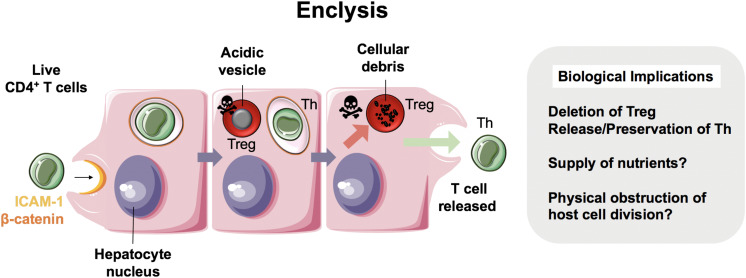
Enclysis in health and in hepatocellular carcinoma. Enclysis is the enclosure and lysis of regulatory T cells (Treg) by hepatocytes and hepatocyte cancer cells (13). We showed that Treg cells were captured preferentially compared to non-regulatory CD4^+^ T cells, and also suffered a different fate, as non-regulatory T cells often survived hepatocyte entry.

We have recently reported a distinct cell capture process within the liver termed *enclysis* ($\text{E}\,\gamma \,\kappa \,\lambda \,\varepsilon \,\,\omega \,\overset{´}{\varepsilon }\,\gamma$- ($\overset{´}{\varepsilon }$v-) + κλεω, to enclose, to confine, to keep in captivity), in which live CD4^+^ T cells are captured by hepatocytes (Figure 4) (13). This process occurred *in vitro*, in primary human hepatocytes and in hepatoma cells (Huh-7 and HepG2 cells), and *ex vivo* within patient liver samples. T cells were also found to reside within hepatocytes *in vivo* as shown in 30 μm-thick sections from cirrhotic patients.

Whilst intercellular adhesion molecule-1 (ICAM-1) facilitated early T cell adhesion to hepatocytes, the ligands for ICAM-1 are not distinct to CD4^+^ T cells and therefore this adhesion molecule does not explain enclysis specificity. Interestingly, adhesion molecule and junctional protein, β-catenin, selectively associated with the enclytic vesicle, in contrast to the efferosome (phagosome containing dead cell) which showed no β-catenin localization. Despite both entosis and enclysis involving formation of membrane blebs (13, 44, 45), enclysis was distinguished from entosis by the lack of E-cadherin association with the enclytic vesicle. Notably, instances of entosis were observed between Huh-7 hepatoma cells, where a clear localization of E-cadherin was apparent. The lack of requirement for the RhoA/ROCK pathway, similar to suicidal emperipolesis, provides a further distinction of enclysis and entosis. Enclysis resembles macropinocytosis, in that there are significant membrane alterations during cell capture events including ruffling, blebs and lamellipodia formation (13, 69), which is in contrast to emperipolesis where these membrane protrusions are absent (63). Furthermore, the wortmannin-insensitivity of enclysis further defines this process as mechanistically distinct, compared with emperipolesis which is abrogated by wortmannin treatment (11).

Whilst CD4^+^ T cells were specifically targeted over CD8^+^ T cells and CD20^+^ B cells, Tregs were three times more likely to be engulfed than non-Treg cells. Vesicles containing Tregs readily acidified with cells undergoing degradation via the lysosomal pathway, unlike non-Tregs, which survived for long periods and remained connected to the extracellular space via the endocytic pathway. Moreover, FOXP3^+^ Tregs were more frequently found within hepatocytes than Tbet^+^ effector cells, in both donor livers surplus to clinical requirement and liver explants from end-stage disease patients (13). Thus, we propose enclysis as a novel immunomodulatory pathway within the liver that could offer therapeutic opportunities to toggle inflammation. But why would hepatocytes possess the ability to target Tregs for degradation when an integral function of the liver is to maintain immunotolerance? Although this seems counter-intuitive, given the previous studies which have evidenced a role for the liver in maintaining peripheral immune tolerance (70), it is conceivable that enclysis could act as a biological switch, preventing the liver from becoming “immunoblind”. The ability of hepatocytes to control local T cell populations and modulate ratios of regulatory and effector cells may represent an intrinsic mechanism by which the liver can rapidly respond to its local inflammatory environment. The stimuli and endogenous regulators of enclysis, however, are yet to be defined.

Identification of selective modulators of enclysis may offer opportunities for therapeutic intervention. On one hand, inhibition of Treg cell capture and/or degradation may be successful in situations where it is desirable to enrich local Treg populations and dampen inflammation. Indeed, research surrounding Treg cell-based therapy is ongoing (71–75), and combination with pharmacological inhibitors of enclysis may show promise for several indications, including chronic inflammation and to promote immunotolerance following organ transplantation. Alternatively, in the context of cancer, boosting Treg sequestration or modulating release of effector T cell subsets may be beneficial to enhance tumor immunogenicity (76).

## Implications and Future Perspectives

The impact of cell-in-cell structures on the host cell biology has only recently been investigated. Phagocytosed cells that enter the phagocyte as apoptotic cells or cellular debris, and also engulfed live cells that may subsequently die inside endosomes, can present an added source of nutrients. However, CIC may have longer-lasting implications on the host cell.

Consequences of viable cell internalization include eventual death of host or target cells, target cell division or release, or prevention of host cell division which can cause multinucleation, polyploidy and aneuploidy (19, 77). This has implications for cancer metastatic potential (78), and links between aneuploidy and genomic instability (loss of tumor suppressor genes) have been established (19, 77). A recent study has shown that p53 mutations in lung adenocarcinoma patients are associated with increased incidence of cell-in-cell structures, and that mutant p53 expression promotes entotic engulfment, tumorigenesis and disease recurrence (51). Whilst host cells lacking p53 had perturbed cell division and subsequent death, mutant p53 cells underwent aberrant cell division, multinucleation, and tripolar mitosis. Thus, p53 expression facilitated pro-tumorigenic entotic engulfment and abnormal mitosis, which consequently contributed to genomic instability.

Cell-in-cell structures in patients are indicative of worse clinical grade and poor prognosis (51, 58, 79). In the context of the liver, ploidy changes and multinucleation in hepatocytes are important considerations for liver regeneration (25–27, 80–82) and associate with various pathological processes (83). In a study where oxidative stress was shown to promote polyploidy in non-alcoholic fatty liver disease, the authors suggested that hepatocyte multinucleation preceded the onset of hepatocellular carcinoma (84). In the absence of cancer, it is now understood that the polyploid state in mice may restrict hepatocyte proliferation and liver regeneration (81).

It is important to consider the biological impact of cell-in-cell structures in liver diseases. Hepatocytes have evolved to eliminate apoptotic and necrotic cells efficiently to prevent inflammation, and this is also true for other CIC processes, the mechanisms of cell death in the liver have been described previously (85, 86). Failure to eliminate necrotic or autoreactive cells would exacerbate liver injury and increase the incidence of fibrosis. Fibrosis (liver scarring) is the consequence of various chronic liver diseases caused by viral, autoimmune, metabolic or cholestatic liver injury, and can lead to cirrhosis and end-stage disease requiring a transplant. The precise mechanism of bile acid hepatotoxicity has not been fully elucidated.

Non-alcoholic fatty liver disease (NAFLD) is of increasing concern at a global scale, and up to 25% of patients can progress to non-alcoholic steatohepatitis (NASH). Increased liver enzymes denote hepatocellular damage [ALT, AST, and others, reviewed in (86)]. The molecular mechanisms controlling hepatocellular injury have begun to emerge in recent studies that revealed a role for the transcription regulator TAZ in preventing hepatocyte death, inflammation and fibrosis (87, 88). Further, hepatocyte Notch activation was linked directly to NASH-related fibrosis (89). The role of efferocytosis in the clearance of apoptotic cells and the prevention of necrotic cell injury and fibrosis in NASH has been reviewed recently (90).

The pro- or anti-inflammatory impact of enclysis in NASH remains to be established, however, NASH liver explants show measurable CD4^+^ T cells inside hepatocytes, including FOXP3^+^ and Tbet^+^ T cells (13). Of note, Ma et al. showed that in NAFLD, dysregulation of lipid metabolism causes a selective loss of intrahepatic CD4^+^ but not CD8^+^ T cells, leading to impaired tumor surveillance and accelerated carcinogenesis (91). The mechanism of CD4^+^ T cell elimination in this context has not been described, however, it was shown that T cells died by apoptosis following linoleic acid exposure from lipid-laden hepatocytes.

## Conclusion

**FIGURE 5:**
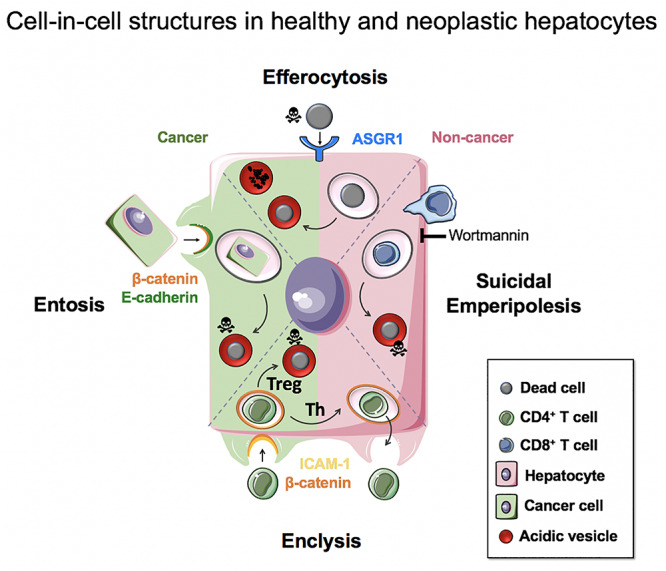
Cell-in-cell structures in healthy and neoplastic hepatocytes. Efferocytosis (apoptotic or necrotic cells), suicidal emperipolesis (autoreactive CD8^+^ T cells) and enclysis (CD4^+^ T cells, Treg) have been reported for non-neoplastic hepatocytes. Neoplastic hepatocytes can also perform efferocytosis and enclysis, and engulf fellow cancer cells that detached from matrix by entosis.

The engulfment of live, apoptotic and necrotic cells by hepatocytes has important implications for their biology in health, inflammation and cancer. These range from nutrient acquisition that can promote cancer cell survival in poorly vascularized tumors, to changes in ploidy that can affect liver regeneration and cancer aggressiveness. It is therefore important to understand the molecular mechanisms that govern these processes so that they can be targeted specifically for patient benefit. Figure 5 summarizes our current knowledge of cell-in-cell structures linked to hepatocyte biology.

Increasing the clearance or necrotic cells is an important goal to prevent inflammation and liver failure, including in catastrophic drug-induced liver injury such as paracetamol (acetaminophen) toxicity. Modulation of T cell capture by suicidal emperipolesis (CD8^+^ T cells) or enclysis (Treg cells) has the potential to influence liver tolerance and toggle inflammation in conditions such as autoimmune hepatitis, viral infection or liver cancer, where the unmet clinical needs are profound. We propose that understanding CIC structure mechanisms will enable specific therapeutic targeting and has the potential to provide new therapeutic targets for liver diseases and liver cancer.

## Author Contributions

LT and ZS prepared the figures. SD, LT, AW, and ZS wrote the review.

## Disclaimer

The views expressed are those of the author(s) and not necessarily those of the NHS, the NIHR or the Department of Health.

## Conflict of Interest

The authors declare that the research was conducted in the absence of any commercial or financial relationships that could be construed as a potential conflict of interest.
